# Supplementary material for: Mechanisms governing the pioneering and redistribution capabilities of the non-classical pioneer PU.1
Source: Nat Commun. 2020 Jan 21;11:402. doi: 10.1038/s41467-019-13960-2 (PMC6972792; doi:10.1038/s41467-019-13960-2)
Supplement: Supplementary file 7 — Source data [file 41467_2019_13960_MOESM7_ESM.zip › Source_Data/FigureS2/FigureS2B_MotifScanOutput/KmeansCluster5/homerResults/motif13.similar.html]

motif13

## Information for motif13

C
T
A
G
T
C
G
A
C
G
A
T
T
G
C
A
C
T
A
G
A
C
G
T
C
A
T
G
G
C
T
A
C
A
T
G
G
C
A
T
  
Reverse Opposite:  

C
G
T
A
G
T
A
C
C
G
A
T
G
T
A
C
G
T
C
A
G
A
T
C
A
C
G
T
G
C
T
A
A
G
C
T
G
A
T
C
  

|  |  |
| --- | --- |
| p-value: | 1e-68 |
| log p-value: | -1.581e+02 |
| Information Content per bp: | 1.587 |
| Number of Target Sequences with motif | 200.0 |
| Percentage of Target Sequences with motif | 5.03% |
| Number of Background Sequences with motif | 384.5 |
| Percentage of Background Sequences with motif | 0.90% |
| Average Position of motif in Targets | 99.1 +/- 40.1bp |
| Average Position of motif in Background | 101.7 +/- 71.7bp |
| Strand Bias (log2 ratio + to - strand density) | -0.1 |
| Multiplicity (# of sites on avg that occur together) | 1.09 |
| Motif File: | file (matrix) reverse opposite |

### Similar de novo motifs found

|  |  |  |  |  |  |  |  |
| --- | --- | --- | --- | --- | --- | --- | --- |
| Rank | Match Score | Redundant Motif | P-value | log P-value | % of Targets | % of Background | Motif file |
| 1 | 0.856 | A C G T C G T A C T A G A G C T A C T G C G T A A C T G A C G T | 1e-27 | -63.334825 | 5.35% | 2.16% | motif file (matrix) |
| 2 | 0.756 | C G T A C G T A C G T A A C T G A G C T A C T G A C G T A C T G | 1e-14 | -32.736454 | 2.96% | 1.26% | motif file (matrix) |
| 3 | 0.802 | C G T A A C T G A G C T C T A G A C G T C T A G A C G T | 1e-13 | -31.166787 | 17.56% | 13.15% | motif file (matrix) |
